# Amyloid beta soluble forms and plasminogen activation system in Alzheimer’s disease: Consequences on extracellular maturation of brain‐derived neurotrophic factor and therapeutic implications

**DOI:** 10.1111/cns.13082

**Published:** 2018-11-06

**Authors:** Francesco Angelucci, Kateřina Čechová, Richard Průša, Jakub Hort

**Affiliations:** ^1^ Memory Clinic, Department of Neurology, 2nd Faculty of Medicine Charles University and Motol University Hospital Prague Czech Republic; ^2^ International Clinical Research Centre St. Anne’s University Hospital Brno Czech Republic; ^3^ Department of Medical Chemistry and Clinical Biochemistry, 2nd Faculty of Medicine Charles University and Motol University Hospital Prague Czech Republic

**Keywords:** Alzheimer’s disease, amyloid beta, brain‐derived neurotrophic factor, plasminogen activator inhibitor‐1, tissue‐type plasminogen activator

## Abstract

Soluble oligomeric forms of amyloid beta (Aβ) play an important role in causing the cognitive deficits in Alzheimer’s disease (AD) by targeting and disrupting synaptic pathways. Thus, the present research is directed toward identifying the neuronal pathways targeted by soluble forms and, accordingly, develops alternative therapeutic strategies. The neurotrophin brain‐derived neurotrophic factor (BDNF) is synthesized as a precursor (pro‐BDNF) which is cleaved extracellularly by plasmin to release the mature form. The conversion from pro‐BDNF to BDNF is an important process that regulates neuronal activity and memory processes. Plasmin‐dependent maturation of BDNF in the brain is regulated by plasminogen activator inhibitor‐1 (PAI‐1), the natural inhibitor of tissue‐type plasminogen activator (tPA). Therefore, tPA/PAI‐1 system represents an important regulator of extracellular BDNF/pro‐BDNF ratio. In this review, we summarize the data on the components of the plasminogen activation system and on BDNF in AD. Moreover, we will hypothesize a possible pathogenic mechanism caused by soluble Aβ forms based on the effects on tPA/PAI‐1 system and on the consequence of an altered conversion from pro‐BDNF to the mature BDNF in the brain of AD patients. Translation into clinic may include a better characterization of the disease stage and future direction on therapeutic targets.

## 1 ALZHEIMER'S DISEASE AND AMYLOID BETA SOLUBLE FORMS

Alzheimer’s disease (AD) is a type of dementia characterized by an age‐related progressive decline in mental ability.^1^ It is characterized by the extracellular accumulation of amyloid beta (Aβ) peptide and intracellular aggregates of protein tau (neurofibrillary tangles). For many years, these pathological processes were considered the main cause of synaptic degeneration and cell death.^2^, ^3^ Accordingly, many treatments aimed at reducing and/or preventing amyloid plaque formation were proposed during these years. However, many clinical trials, based on information from AD animal models, were not effective in human AD patients. For these reasons, the amyloid cascade hypothesis has been modified. In details, it was observed that the accumulation and deposition of Aβ in the brain is not sufficient to cause the disease as healthy subjects may also present plaques morphologically similar to those of AD patients.^4^, ^5^

Recent studies have shown that soluble forms of Aβ and tau proteins could be more toxic than aggregated forms.^6^, ^7^, ^8^ This mechanism is still not clear, but it has been hypothesized that soluble forms may target specific synaptic sites and disrupt synaptic signaling pathways, thus altering memory functions and causing at last the death of the neuron.^8^, ^9^, ^10^, ^11^ It has been proposed that the plaques represent an initial attempt to sequester soluble forms from extracellular space and preclude their toxicity.^12^ Later on, when the sequestering capacity of the plaques is reduced once they become saturated, soluble forms are free to diffuse in the synaptic space and to bind synaptic sites, causing AD memory deficits.^13^ This hypothesis has been supported by electrophysiological data showing that soluble Aβ forms may alter long‐term potentiation (LTP) and thus memory processes in hippocampal neurons.^14^, ^15^, ^16^ Moreover, it was shown that soluble Aβ42 oligomers taken from the brain of AD patients affect hippocampal function in rodents by enhancing long‐term depression (LTD), inhibiting LTP, and decreasing the number of synapses.^14^

These findings have encouraged the researchers to focus on the identification of the neuronal pathways targeted by soluble forms, in the attempt to develop new therapeutic approaches.^17^ One of the possible pathways explored in this review is represented by the neurotrophin brain‐derived neurotrophic factor (BDNF) and the process that leads to the maturation from the pro‐form (pro‐BDNF) to the biologically active form (BDNF). This process in brain neurons is regulated extracellularly by enzymes of the plasminogen activation system.

## 2 THE PLASMINOGEN ACTIVATION SYSTEM IN THE CENTRAL NERVOUS SYSTEM

The plasminogen activation system is a system composed by different enzymes that control the synthesis of plasmin. The urokinase plasminogen activator (uPA) and the tissue‐type plasminogen activator (tPA) are the enzymes that activate the process of transformation from plasminogen to plasmin. Their activity is in turn regulated by PA inhibitor type 1 (PAI‐1) and type 2 (PAI‐2) and nexin.^18^ In the brain, it has been demonstrated that activation of plasmin is typically dependent on the tPA/PAI‐1 enzymes.

Outside the CNS, the plasminogen activation system is implicated in fibrinolytic mechanisms because the substrate of plasmin is fibrin.^19^ However, tPA can be expressed by cellular elements of the CNS, including neurons, astrocytes, oligodendrocytes, and microglia.^20^, ^21^ This suggests that tPA might be involved in many functions within the brain.^21^

During development, tPA has a role in neuronal migration and synaptic outgrowth, while in the adult brain tPA can modulate neurotransmission, synaptic plasticity, and cognitive functions.^22^, ^23^, ^24^ In adult neurons, tPA is synthesized and stored in vesicles^25^, ^26^ and it is released extracellularly upon neuronal depolarization.^27^, ^28^ Both intracellular tPA protein and mRNA are localized to the synapse and tPA transcription is regulated in an immediate‐early manner.^27^ Once in the extracellular space, tPA can convert plasminogen into plasmin and this proteolytic cascade is counteracted by the expression of the inhibitors, PAI‐1 and neuroserpin. Accordingly, the tPA–plasmin axis has been implicated in several neuronal activities, including LTP,^29^ LTD,^30^ NMDA receptor‐mediated signaling,^31^ and synaptic remodeling.^32^ These data suggest that tPA and its inhibitor PAI‐1 may play a role in synaptic modulation in normal as well as in pathological conditions, such as those occurring during the course of AD.

## 3 THE PLASMINOGEN ACTIVATION SYSTEM IN ALZHEIMER'S DISEASE

Several data in AD animal models and in AD patients indicate a possible involvement of the plasminogen activation system in AD.^33^, ^34^, ^35^, ^36^, ^37^, ^38^, ^39^, ^40^, ^41^, ^42^, ^43^, ^44^, ^45^, ^46^, ^47^ These data mainly show altered levels and/or activity of the most relevant components of this system: plasmin, tPA, and PAI‐1 enzymes (Table 1).

**Table 1:** Main findings on the plasminogen activation system in Alzheimer’s disease

Tissue	Species	Result	References
Neuronal cultures	Rat, Mouse	Plasmin degrades Aβ	33, 34, 36
Neuronal cultures	Rat	Plasmin protects neurons from Aβ‐induced cell death	33, 34
Brain homogenates	Human	Plasmin reduced level and activity in AD	35, 37, 38
Brain	Mouse	Plasmin reduced level and activity in AD	39
Brain	Human	tPA reduced activity in AD	41
Brain	Human	tPA negatively correlates to Aβ levels in AD	42
Brain	Human	tPA protein levels are unchanged or increased in AD	41, 42
Brain	Mouse	PAI‐1 levels increased in AD models	39, 40
Brain	Human	PAI‐1 increased in AD	44
Plasma	Human	PAI‐1 increased in MCI	45
Brain	Mouse	PAI‐1 increases Aβ accumulation during aging	46
Plasma	Human	PAI‐1 levels increases in AD as dementia progresses	47

AD: Alzheimer’s disease; Aβ: amyloid beta; PAI‐1: plasminogen activator inhibitor‐1; tPA: tissue‐type plasminogen activator.

In neuronal cultures, plasmin is capable to cleave, degrade, and reduce both non‐aggregated monomeric and aggregated fibrillar Aβ forms.^33^, ^34^, ^35^ In addition, plasmin protects these neurons from Aβ‐induced cell death^33^, ^34^ and enhances clearance of Aβ in AD animal models when PAI‐1 is pharmacologically inhibited.^36^ These findings suggest that the protease activity of plasmin may be altered during AD. Indeed, it has been shown in AD human brain homogenates that plasmin activity is reduced as compared to that of normal subjects.^37^ In addition, brain tissue (hippocampus and neocortex) homogenates from AD patients have reduced plasmin levels.^38^ Nonetheless, in other studies, it was shown that plasminogen and plasmin protein levels were not significantly altered in frontal and temporal cortex homogenates from AD patients.^35^

As for plasmin, there are data indicating that tPA and PAI‐1 can be altered in AD. tPA has been shown to be highly expressed in the brain areas where plaques are deposited. Nonetheless, in AD animal models where endogenous tPA was genetically reduced, a greater accumulation of Aβ was observed, in association with synaptic dysfunction and memory deficits.^39^ In addition, it was shown that tPA‐mediated plasmin activity declines throughout the brain, causing Aβ deposition during aging.^40^ Similarly, in humans, it was found that tPA activity is reduced in the brain of AD patients as compared to controls^41^ and negatively correlates to Aβ levels,^42^ while tPA protein levels are unchanged^41^ or increased.^43^

Conversely, it has been shown that PAI‐1 is elevated in AD. This increase has been reported in the brain of animal models^39^, ^40^ and in humans.^44^, ^45^ In addition, in AD mice, it was shown that PAI‐1 expression and activity contribute to Aβ accumulation during aging, a phenomenon that can be probably attributed to the inhibition of plasminogen activation and to the related reduction of Aβ degradation.^46^ These findings have been paralleled by data in plasma of AD patients where PAI‐1 levels were found increased (as dementia progressed) and correlated with the decline in cognitive function.^47^

## 4 MECHANISM OF ACTION OF Aβ ON THE PLASMINOGEN ACTIVATION SYSTEM

As stated before, tPA and PAI‐1 are produced by elements of the CNS, including neurons and glial cells. The information gained from AD animal models indicate that, as the level of Aβ increases, there is a concomitant overproduction of PAI‐1 and a decrease in tPA/plasmin activity. Regarding the possible mechanism, it has been suggested that the reduction of tPA activity, which can cause a greater Aβ accumulation, is due to the overproduction of PAI‐1 by CNS cells rather than to a direct effect of Aβ. In fact, increased amount of Aβ peptides during AD course do not affect, or even increase, the protein levels of brain tPA (and plasmin). On the opposite, it has been suggested that Aβ can stimulate, directly or indirectly, PAI‐1 expression in neuronal and glial cells. Supporting this notion, it was found that increased PAI‐1 levels are present in regions where Aβ accumulates, such as cerebral cortex, but not in other brain Aβ‐free areas.^46^ In some rodent studies,^42^, ^48^ it was found that overproduction of PAI‐1 mainly occurs in the presence of gliosis associated with Aβ load. As astrocytes are a main source of PAI‐1,^49^ the authors suggest that astrogliosis induced by Aβ may be responsible for the increase in PAI‐1. The presence of pro‐inflammatory cytokines, such as tumor necrosis factor alpha (TNF‐α), may also concur to stimulate PAI‐1 expression.^50^ Alternatively, we cannot rule out the possibility that Aβ may directly stimulate PAI‐1 expression by neurons. One immunohistochemistry study, performed to localize PAI‐1 in the CNS, found a positive immunostaining not only in astrocytes but also in rat and human neurons, with greater neuronal expression in the presence of inflammatory processes^51^, ^52^ which are common in the brain of AD patients.^53^ Furthermore, it has been shown that direct injection of Aβ into brain regions may cause an increase of PAI‐1. Injection of Aβ 1‐40 into CA1 region of the hippocampus of mice lacking tPA or plasminogen causes a strong PAI‐1 accumulation^48^ but not in the wild type. In addition, primary cortico‐hippocampal cultures from mouse AD model (Tg2576) cultured in the presence of the pathogenic fragment Aβ25‐35 immediately show a dramatic increase in PAI‐1 synthesis^54^ through activation of JNK‐dependent c‐Jun pathways.^55^, ^56^

Altogether these data indicate that the changes in the expression of plasminogen activators and inhibitors may occur during AD course and account, at least in part, for Aβ accumulation and lack of degradation. However, these proteins have other actions that may be relevant in AD. Among the events linked to the expression of these enzymes, there is also the regulation of gene expression of neurotransmitters and other proteins involved in the regulation of synaptic function and in the survival of neurons affected during AD. Among these proteins, the neurotrophin brain‐derived neurotrophic factor (BDNF) has generated considerable interest because of the effects that altered levels of this protein may have on CNS neurons. Since the demonstration that the conversion from pro‐BDNF to mature BDNF is regulated by plasminogen activation system, the link between this system and BDNF metabolism has been investigated.

## 5 PLASMINOGEN ACTIVATION SYSTEM AND THE REGULATION OF BDNF MATURATION

In the CNS, BDNF maturation is dependent on tPA/plasmin system. Plasmin activates the extracellular conversion from pro‐BDNF to the mature form^57^ and this process is regulated by PAI‐1.^58^ At the same time, plasmin activity in the brain is typically dependent on tPA.^59^ Thus, tPA/PAI‐1 system represent an important regulator of extracellular BDNF/pro‐BDNF ratio.^60^

It has been shown that exogenous tPA administration increases hippocampal BDNF levels^61^ and the conversion of pro‐BDNF to BDNF by plasmin is essential for LTP late‐phases.^62^, ^63^ In addition, a defective tPA/plasmin/PAI‐1‐mediated BDNF maturation has been claimed to be involved in the manifestation of some brain pathologies, such as substance abuse and addiction,^64^, ^65^ depression,^66^ and stress.^67^

These data are not surprising given the fact that pro‐BDNF and BDNF may have opposite effects on survival and function of CNS neurons.

## 6 ROLE OF BDNF AND pro‐BDNF IN THE CNS

Brain‐derived neurotrophic factor is the most abundant neurotrophin in the brain and is present and utilized in many brain regions, including cortex, hippocampus, striatum, hypothalamus, and cerebellum.^68^ It has been established that BDNF mediates survival and differentiate activities on neurons by binding and activating the tropomycin receptor kinase B (TrkB).

BDNF is synthesized as a precursor (pro‐BDNF)^69^ which is cleaved by hormone convertases^70^, ^71^ or by plasmin extracellularly to release the mature form.^72^ The pro‐BDNF was initially described as an inactive precursor. However, it was lately shown that pro‐BDNF acts as independent ligand activating the p75 receptor, rather than TrkB.^73^, ^74^ The p75 receptor is a member of the tumor necrosis factor family that encodes a cytoplasmic apoptotic death domain. The p75 receptor binds to the mature domain region of pro‐BDNF.^73^, ^75^ Indeed, treatment of neurons that express p75 with recombinant pro‐BDNF induces cell death.^73^

In neurons, transcription, processing, and secretion of BDNF are regulated by synaptic activity.^76^ This fact has generated the idea that BDNF may regulate activity‐dependent forms of synaptic plasticity, such as for example LTP.^77^ On the other hand, there are indications that the activation of pro‐BDNF through the p75 receptor may produce opposite effects on hippocampal neurons. In pro‐BDNF−expressing mice, it has been shown that pro‐BDNF negatively regulates hippocampal dendritic complexity and spine density. In addition, hippocampal slices from these mice display reduced synaptic activity and enhanced LTD.^78^ Expression of pro‐BDNF may also elicit additional effects, such as growth cone retraction,^75^ axonal pruning,^79^ LTD induction,^80^, ^81^ and synaptic elimination of neuromuscular junctions.^82^ These effects contrast with those elicited by BDNF, leading to the “yin‐yang” neurotrophin hypothesis in which mature BDNF and pro‐BDNF exhibit opposing functions mediated by the activations of their respective receptors, TrkB and p75.^60^, ^83^

These data suggest that the conversion from the pro‐BDNF to the mature BDNF is an important process that regulates hippocampal activity and memory processes. Thus, alteration of the BDNF/pro‐BDNF rate of conversion may have relevance to several brain pathologies, including neurodegenerative disorders such as AD.^84^, ^85^, ^86^

## 7 DATA ON BDNF AND pro‐BDNF IN AD

Brain‐derived neurotrophic factor expression has been investigated in the brain and serum of AD patients (Table 2). Many studies showed that AD patients exhibit altered BDNF levels in the brain^87^ and blood,^88^, ^89^, ^90^ and conversely, several animal studies demonstrate a potential protective effect of BDNF against Aβ‐induced neurotoxicity.^72^, ^91^

**Table 2:** Main findings on BDNF in Alzheimer’s disease patients

Tissue	Species	Result	References
Serum	Human	Decreased BDNF levels in AD	87, 89, 92, 93, 94, 95
Serum	Human	Decreased BDNF levels in MCI	87, 92
Serum	Human	Increased or unchanged BDNF levels	88, 89, 97
CSF	Human	Decreased BDNF levels in MCI and AD	94, 98, 100
CSF	Human	Unchanged BDNF levels in AD	90
Brain	Human	Reduced BDNF mRNA and protein levels in AD	101, 102, 103
Brain	Human	Reduced pro‐BDNF levels	104, 105, 106
Brain	Human	Reduced TrkB receptor immunoreactivity in AD	107
Brain	Human	No changes in TrkB receptor immunoreactivity in AD	108, 109

AD, Alzheimer’s disease; BDNF, brain‐derived neurotrophic factor; CSF, cerebrospinal fluid; MCI, mild cognitive impairment; TrkB, tyrosine kinase receptor B.

In serum, many studies have reported a reduction of BDNF levels in AD patients as compared to healthy subjects.^87^, ^89^, ^92^, ^94^, ^95^ In addition, patients with mild cognitive impairment (MCI) may also be characterized by decreased BDNF levels as compared to controls.^87^, ^93^ Furthermore, decreased levels of BDNF in MCI and AD patients seem to be associated with hippocampal structural changes and decrease in cognitive performance.^87^, ^93^, ^96^ Despite these evidences, there is still a lack of consensus about BDNF profile in AD patients. Other studies have in fact reported either increased BDNF levels in both MCI and AD patients^88^, ^89^ or no difference between AD and controls.^97^

Likewise, studies on cerebrospinal fluid (CSF) BDNF levels in AD and other dementias report similar results. Forlenza et al^98^ found decreased BDNF concentration in CSF, associated with faster progression from amnestic MCI to AD. Blasko et al^99^ did not find a significant difference in BDNF between AD and controls, while Laske et al^94^ showed lower CSF BDNF levels among AD patients in comparison with healthy controls and non‐AD dementia patients. Furthermore, a study in healthy older adults showed an association of lower CSF BDNF levels with poorer memory performance and faster cognitive decline.^100^

In the brain of AD patients, it was found that both BDNF mRNA and protein levels are reduced in hippocampus and temporal cortex^101^, ^102^ while protein levels are reduced in the hippocampus and the parietal cortex.^103^ Other studies have shown that the pro‐BDNF is also reduced in parietal cortex^104^ and in nucleus basalis of Meynert (nbM) of AD patients^105^ and this decrease precedes the decline in choline acetyltransferase activity.^103^, ^106^ To the best of our knowledge, there are no data on pro‐BDNF measurement in the serum or CSF of AD patients. There are also data indicating changes in BDNF receptor in the brain of AD patients. One study showed a consistent reduction of TrkB immunoreactive neurons in the nbM of AD patients^107^ while other studies reported no changes in TrkB receptor.^108^, ^109^

These data have generated the consensus that in the brain of AD patients, there is a lack of BDNF support to neurons, which can contribute to synaptic dysfunction, cognitive decline, and lately to neuronal death. Nonetheless, the mechanism by which aggregated or soluble Aβ forms interfere with BDNF activity in neurons is not yet clear. In the following paragraph, we will describe a possible pathogenic mechanism caused by soluble Aβ forms based on the effects on tPA/PAI‐1 system and, as illustrated previously, the consequence of an altered conversion from pro‐BDNF to the mature BDNF.

## 8 PATHOGENIC MECHANISM OF Aβ SOLUBLE FORMS IN AD THROUGH CHANGES IN TPA, PAI‐1, AND BDNF

Synaptic dysfunction plays an important role in AD pathophysiology. As said before, the most recent hypotheses in AD involve soluble forms of Aβ, which play an important role in causing cognitive deficits by specifically targeting synaptic sites and disrupting signaling pathways.^10^ Nonetheless, the molecular details of this process have not been fully elucidated.

**Figure 1:**
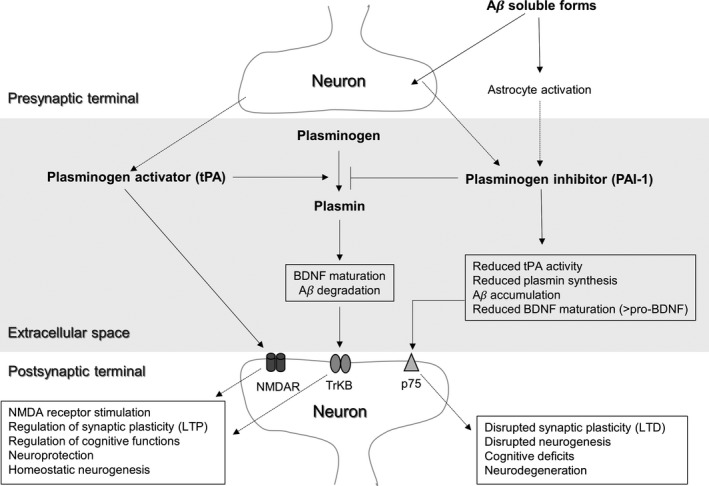
Relationship between plasminogen activation system and BDNF at the synaptic level. tPA and PAI‐1 may be released into the extracellular space where plasminogen is also present. tPA can influence synaptic activity by two ways: (1) by binding directly to specific subunits of NMDA receptors and (2) by increasing the levels of plasmin, which in turn leads to increased BDNF maturation and increased synaptic activity (LTP) after binding with the TrkB receptor. PAI‐1 inhibits tPA activity, thus reducing the synthesis of plasmin and the maturation of BDNF. In the context of Alzheimer‘s disease, elevated PAI‐1 levels may account for increased Aβ accumulation and increased pro‐BDNF, which reduces synaptic function (LTD) after binding with the p75 receptor. Long‐term consequences may include cognitive deficits and fostering of neurodegenerative processes

The data exposed above suggest that one of the possible mechanisms by which soluble Aβ forms affect synaptic function is linked to the changes observed in the tPA/PAI‐1 system and, consequently, to the effects on pro‐BDNF/BDNF ratio (Figure 1). Supporting this hypothesis, a recent study^54^ with a mouse genetic model of familial AD (Tg2576) and post‐mortem brain tissues has shown that Aβ soluble forms might impair BDNF proteolytic processing through modulation of neuronal PAI‐1.

**Figure 2:**
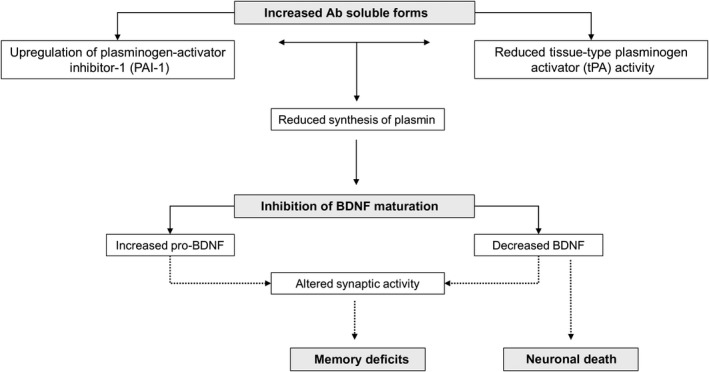
Putative scheme of pathogenic mechanism of amyloid beta soluble forms in AD through tPA/PAI‐1 and BDNF. By upregulating PAI‐1, Aβ soluble forms inhibit tPA and plasmin synthesis. This mechanism impairs the extracellular conversion from pro‐BDNF to the mature BDNF. An altered ratio of pro‐BDNF/BDNF in favor of the pro‐form may lead to an enhancement of LTD and reduction of LTP in hippocampal neurons, with consequent synaptic dysfunction and memory deficits. In addition, a long‐lasting reduction of mature BDNF may contribute to neuronal atrophy and death. Aβ: amyloid beta; BDNF: brain‐derived neurotrophic factor; PAI‐1: plasminogen‐activator inhibitor‐1; tPA: tissue plasminogen‐activator

Thus, one plausible hypothesis is that elevated levels of PAI‐1 in AD inhibit the synthesis of plasmin by tPA. The consequences of this inhibition in the brain may be multiple. First, a reduced synthesis of plasmin may contribute to Aβ accumulation in the brain and thus accelerate the disease process. Second, a reduced plasmin synthesis may lead to reduced extracellular conversion of pro‐BDNF to BDNF. An altered ratio of pro‐BDNF/BDNF in favor of the pro‐form may lead to an enhancement of LTD and to a reduction of LTP in hippocampal neurons, with consequent synaptic dysfunction and memory deficits. Lastly, a long‐lasting reduction of mature BDNF, which is the most important neurotrophic factor in the CNS, may contribute to neuronal atrophy and ultimately to neuronal death. A scheme summarizing this mechanism is represented in Figure 2.

## 9 DIAGNOSTIC AND THERAPEUTIC IMPLICATIONS IN AD

Based on the data presented, there are some important considerations from both diagnostic and therapeutic points of view.

The therapies available for AD treatment are only able to slow down but not to stop the disease progression. In this scenario, the prevention and an early diagnosis are important tools to increase the quality of life of these patients and to reduce the costs to the families and to the public healthcare system. Additional use of biomarkers could make the AD diagnosis easier and faster, as already demonstrated for Aβ 1‐42 and tau in CSF.^110^

The data presented here suggest that measurement of BDNF/pro‐BDNF ratio and tPA/PAI‐1 ratio in biological fluids (serum, plasma, CSF), in association with neuroimaging data and neuropsychological characterization, may serve as indirect indicators of the “synaptic state” in the brain of AD subjects. If a direct connection between these biomarkers and cognitive decline could be established, there will be the possibility to treat the patients according to their risk of developing severe or moderate forms of dementia. Testing these protein levels in blood is a quite simple procedure, without risk for the patient, conventionally used in most of laboratory analysis. Notably, the strategy of combined measures of tPA‐BDNF pathway proteins in serum (including pro‐BDNF) has been recently adopted in subjects affected by different mental disorders. The results have shown that the combined measures of protein levels of the tPA‐BDNF pathway were better than single protein measurement, in terms of accuracy of diagnosis and differentiation of these disorders.^66^, ^111^

In addition, investigation of proteins involved in synaptic regulation and neuronal survival, such as BDNF and the related proteins described here, will give a significant contribution to the understanding of pathophysiology of AD and an incentive to develop therapeutic strategies based on the targeting of elements of the pathway involved. The modulation of brain BDNF (and other related trophic factors) has already been investigated as potential treatment strategy in a large group of CNS disorders^112^, ^113^ including AD and Parkinson’s diseases, amyotrophic lateral sclerosis, Huntington’s disease, and peripheral neuropathy. Most of the trials, however, have failed because of the difficulty of these proteins to cross the blood–brain barrier and to deliver the right amount of trophic factor in the target region. Instead, the tPA/PAI‐1 pathway can be targeted by pharmacological agents,^54^ a strategy that has been already adopted in other types of diseases such as diabetic nephropathy^114^ and fibrosis.^115^ However, there is a need to explore the effects of these agents on BDNF expression in healthy and AD subjects.

Interestingly, a recent study has shown that administration of a PAI‐1 inhibitor for a period of 6 weeks reduces Aβ load in the hippocampus and cortex and improve learning and memory function in an AD mouse model.^116^ Notably, these effects were associated with increased tPA and plasmin activities. Furthermore, in a multiple sclerosis mouse model, it was shown that oral administration of another PAI‐1 inhibitor (TM5484) with high capacity to penetrate the blood–brain barrier was able to up‐regulate gene expression of BDNF and choline acetyltransferase, a marker of cholinergic neuronal density.^117^ These effects were associated to reduced demyelination and axonal degeneration. In another in vitro study, it was shown that PAI‐1 inhibitors are able to reduce pro‐BDNF levels in hippocampal slices from epileptic mice.^118^ These data suggest that the compounds targeting tPA/PAI‐1 pathway, such as PAI‐1 inhibitors, may represent a new strategy to modulate brain BDNF expression in pathological conditions, including AD.

## 10 ADDITIONAL CONSIDERATIONS AND LIMITATIONS

In this review, we have hypothesized that Aβ soluble forms may impair PAI‐1/tPA pathway in the CNS and consequently cause effects on the conversion of pro‐BDNF to the mature BDNF. Despite the evidences provided, we should be aware that there is at present no conclusive evidence that this is the sole mechanism occurring in the brain of AD patients. Thus, we cannot exclude the possibility that PAI‐1 may increase independently from the effect of Aβ and that Aβ itself accumulates because of high PAI‐1 expression/activity. It is likely that the two effects are interconnected and sustain each other with a vicious circle. In addition, there is a body of evidence indicating that Aβ might be involved in the decrease of BDNF in AD. In rat models, injection of Aβ reduces BDNF content in brain regions such as frontal cortex and amygdala.^119^ Moreover, neuroblastoma cells and dendritic cells derived from AD patients cultured in the presence of Aβ display down‐regulation of BDNF protein and mRNA.^120^, ^121^ It has been suggested that Aβ may interfere with BDNF synthesis by acting on the transcription factor‐cyclic adenosine monophosphate (cAMP) response element‐binding protein (CREB) binding to the promoter region of BDNF, leading to decreased BDNF transcription.^120^, ^122^ Alternatively, the structural changes induced by Aβ at the levels of microtubules interfere with BDNF axonal transport.^123^, ^124^ On the other hand, there are also consisting evidences for the neuroprotective effect of BDNF against Aβ‐induced neurotoxicity. Exogenous application of BDNF reduced Aβ production in primary neurons and in the brain of wild‐type mice in vivo.^125^ In addition, BDNF is able to provide neuroprotection and improve learning and memory deficits induced by previous Aβ administration.^126^

In any case, the interconnection among the effects of Aβ, BDNF and the plasminogen activation system suggests that, independently from its origin, this mechanism might be responsible for many clinical features of AD and that a pharmacological treatment targeting elements of this circuit, such as for example PAI‐1, is likely to produce a cascade effect.

## 11 CONCLUSIONS

Aβ soluble forms have been claimed to be responsible for AD pathological symptoms by specifically targeting synapses and disrupting synaptic signaling pathways. Recent data have suggested that one of the targets of Aβ soluble forms may be the enzymatic elements of the plasminogen activation system. The direction of changes of these enzymes points to a down‐regulation of tPA and to an up‐regulation of its inhibitor PAI‐1.

In this review, we have explored the data supporting the hypothesis that the process of conversion of BDNF, a strong regulator of synaptic activity in brain neurons, from the immature form pro‐BDNF to the mature form may be altered by tPA/PAI‐1 changes in AD. By up‐regulating PAI‐1, Aβ soluble forms may impair this conversion and cause an altered extracellular BDNF/pro‐BDNF ratio. The combined effects of Aβ soluble forms on BDNF and tPA/PAI‐1 may account for many AD symptoms, including synaptic dysfunctions, memory deficits, and neuronal death. Therefore, a pharmacological approach based on the targeting of tPA/PAI‐1 system could produce beneficial effects on AD symptoms, possibly by modulation of BDNF gene expression.

## CONFLICT OF INTEREST

The authors declare no conflict of interest.
